# Wounded Transferred to *Dreadnought* Hospital: Excellence of the Navy Transport System

**Published:** 1914-11-07

**Authors:** 


### THE NAVAL RED CROSS TRAIN IN LONDON.

### WOUNDED TRANSFERRED TO "DREADNOUGHT" HOSPITAL.

### Excellence of the Navy Transport System.

THE DIRECTOR-GENERAL'S CONGRATULATIONS.

The Admiralty have sent 102 wounded men from the war to the *Dreadnought* Hospital at Greenwich, where they arrived on Thursday evening, October 29. The significance of this is exceptional in the case of the *Dreadnought* Hospital (since this is understood to be the first time in its history that its aid has been invoked by the Admiralty), existing as it has done for the men of the mercantile marine. It is, however, to be noted that the patients are Army men. These men came from Plymouth in the Naval Red Cross train, and more than half of them are grievously injured and lying down, while many of the so-called sitting-up cases had wounds in the lower limbs which necessitated their being carried.

In order to transport these patients from the train to the hospital the Port of London Authority placed at the disposal of the Seamen's Hospital Society two of their electric ambulances, while eight other ambulances were obtained from the Metropolitan Asylums Board. Some friends of the hospital lent their motor-cars to convey those who were able to sit up, and in this way the entire train-load of 102 men, sixty of whom had to be borne on stretchers, was transferred to the hospital in less than one hour. The lying-down cases were got to bed immediately, and about forty of those who could walk had their wounds dressed in the casualty department before going to the wards. The whole of the patients were in bed within two hours of the train reaching Greenwich Station.

#### Peculiarities of the Naval Train.

To those unfamiliar with the Naval Red Cross train it may be interesting to say a few words on its formation. This particular train is made up of nine long coaches—one a store, one affording accommodation for officers and nursing staff, one for kitchen and other domestic requirements, while each of the remaining coaches is fitted to carry sixteen men lying down in swinging hammocks, or twenty-five sitting down. The inside of each coach may be entirely cleared of every item of furniture, thus rendering it particularly easily disinfected. The cots, as befit the Navy, are of white scrubbed canvas, while white curtains of lighter material run all round the inside of every coach, and so are available to screen the windows. The cots which hang in the train are interchangeable with the cots that hang on shipboard. Hence it is that wounded transported by the Navy are not necessarily moved from the bed in which they are placed on leaving France until the patient is transferred to the ambulance taking him to the hospital bed. In this respect the Naval trains vary considerably from the arrangements for transport in the Military Red Cross railway carriages.

The particular train which brought the patients to the *Dreadnought* Hospital was in charge of a Staff Surgeon, R.N.R., and an Inspector-General of Fleets also accompanied the train from Plymouth. It is a coincidence that the officer who transferred the patients from the hospital ship to the train was Major Kenneth Steele, R.A.M.C., who for so many years was associated with the Seamen's Hospital Society. He held the post of honorary anæsthetist at the *Dreadnought* Hospital, having previously served as house surgeon at both hospitals and surgeon at the East India Dock Dispensary. He is son of the late Dr. Steele, of Guy's, who was known so well a quarter of a century ago as one of the pioneers of modern hospital administration. Probably this is the most serious train-load of wounded that has yet arrived in London. It is most gratifying to all at the *Dreadnought* that a communication has been received from the Director-General intimating that a very fine evolution in transporting patients from train to hospital has been made, and that he congratulates all concerned in the matter.

